# The Effectiveness of Deep Brain Stimulation in Dystonia: A Patient-Centered Approach

**DOI:** 10.5334/tohm.69

**Published:** 2020-06-08

**Authors:** Hendriekje Eggink, Rivka F. Toonen, Jonathan C. van Zijl, Martje E. van Egmond, Anna L. Bartels, Rick Brandsma, M. Fiorella Contarino, Kathryn J. Peall, J. Marc C. van Dijk, D. L. Marinus Oterdoom, Martijn Beudel, Marina A. J. Tijssen

**Affiliations:** 1 Expertise Center Movement Disorders Groningen, Department of Neurology, University Medical Center Groningen, University of Groningen, Groningen, NL; 2 Department of rehabilitation, University of Groningen, University Medical Center Groningen, Groningen, NL; 3 Ommelander Ziekenhuis Groningen, Department of Neurology, Delfzijl and Winschoten, NL; 4 Haga Teaching Hospital, Department of Neurology, The Hague, NL; 5 Leiden University Medical Center, Department of Neurology, Leiden, NL; 6 Neuroscience and Mental Health Research Institute, Division of Psychological Medicine and Clinical Neuroscience, Cardiff University, Cardiff, UK; 7 Department of neurosurgery, University of Groningen, University Medical Center Groningen, Groningen, NL

**Keywords:** Deep brain stimulation, dystonia, goal, patient-centered outcomes, daily functioning

## Abstract

**Background:**

To systematically evaluate the effectiveness of deep brain stimulation of the globus pallidus internus (GPi-DBS) in dystonia on pre-operatively set functional priorities in daily living.

**Methods:**

Fifteen pediatric and adult dystonia patients (8 male; median age 32y, range 8–65) receiving GPi-DBS were recruited. All patients underwent a multidisciplinary evaluation before and 1-year post DBS implantation. The Canadian Occupational Performance Measure (COPM) first identified and then measured changes in functional priorities. The Burke-Fahn-Marsden Dystonia Rating Scale (BFMDRS) was used to evaluate dystonia severity.

**Results:**

Priorities in daily functioning substantially varied between patients but showed significant improvements on performance and satisfaction after DBS. Clinically significant COPM-score improvements were present in 7/8 motor responders, but also in 4/7 motor non-responders.

**Discussion:**

The use of a patient-oriented approach to measure GPi-DBS effectiveness in dystonia provides an unique insight in patients’ priorities and demonstrates that tangible improvements can be achieved irrespective of motor response.

**Highlights:**

- Functional priorities in life of dystonia patients and their caregivers vary greatly
- The effect of DBS on functional priorities did not correlate with motor outcome
- Half of the motor ‘non-responder’ patients reported important changes in their priorities
- The effect of DBS in dystonia should not be measured by motor outcome alone

## Introduction

Dystonia is a movement disorder characterized by sustained or intermittent muscle contractions causing abnormal, often repetitive movements, abnormal posturing, or both. Dystonia comprises a heterogeneous patient population due to a broad spectrum of underlying acquired and inherited etiologies [1].

Over the past decades, deep brain stimulation of the globus pallidus internus (GPi-DBS) has emerged as a safe treatment option with a good response in non-lesional, mostly isolated forms of dystonia and a more variable response in combined forms of dystonia that are due to a static lesion or neurodegenerative process [2]. The application of this elective neurosurgical procedure therefore frequently gives rise to discussion, especially in secondary dystonia patients.

The effect of GPi-DBS has been predominantly measured with objective standardized dystonia rating scales [2]. However, the variability of dystonic symptoms within days, or even hours or minutes, makes it difficult to reliably capture overall dystonia severity in just one evaluation. Furthermore, it is unclear how dystonia severity reflects disease burden and there is only weak evidence that a reduction in symptoms in isolated forms of dystonia may correlate with meaningful improvements in functioning [3,4].

In line with the World Health Organization guidelines advocating patient-centered outcome measures [5], we aimed to systematically evaluate the effect of DBS in terms of individualized functional priorities set by the patient and/or their caregivers.

## Methods

### Patients

We prospectively included fifteen consecutive dystonia patients that received GPi-DBS between January 2013 and July 2016. All patients were evaluated pre and 1-year post-operatively screened by a multidisciplinary team. The local ethical committee classified the study as care as usual.

### Outcome measures

Priorities were identified by the Canadian Occupational Performance Measure (COPM). The COPM is an individualized outcome measure to capture everyday problems that impact daily functioning. Together with a trained occupational therapist, patients and/or caregivers imaginary walked through a typical day in the patient’s life to identify priorities that they would like to see improved by GPi-DBS. For the three most important priorities performance (1–10) and satisfaction (1–10) were rated. Change between pre- and postoperative ratings was used for further analyses. At the 1-year follow-up, patients and/or their caregivers were blinded for their pre-operative ratings. A difference of two or more points was considered clinically significant [6].

Dystonia severity was assessed with the motor subscale of the Burke-Fahn-Marsden dystonia rating scale (BFMDRS). Videos were blinded for operative status and rated by experienced clinicians (ALB, RB, KJP, MFC) who were blinded to treatment state. Mean total scores were calculated. In order to be able to compare the results in all patients (generalized and focal/segmental) the relative change in BFMDRS (% of improvement) was used for further analyses. In addition, patients were subdivided into motor ‘responders’ (>20% change in BFMDRS score) and ‘non-responders’ (<20% change in BFMDRS score) [7]. For absolute scores, see supplementary Table 1.

### Data-analysis

Data-analysis was performed using Statistical Package for the Social Sciences (SPSS, version 23.0). Due to the heterogeneity of the sample, medians and interquartile ranges (IQR) were used. Differences between pre- and postoperative scores were compared with the Wilcoxon Signed Ranked Test for total group and the responders and non-responder subgroups. Correlations between the outcome measures were calculated with the Spearman’s ρ.

## Results

Baseline characteristics, etiology and pharmacological treatment of all 15 patients (8 male; median age 32y range 8–65; median disease duration 8y range 3–47) are shown in Table 1.

**Table 1:** Patient characteristics and pharmacological treatment.

Pt	Gender/age (yr)	Body distribution	Isolated or combined	Etiology	Pre-operative medical treatment	Post-operative medical treatment

1	M/8	Generalized	Combined (spasticity)	Mitochondrial disorder	Gabapentin 100 mg; intrathecal baclofen 3 ug/hr	Unchanged
2	M/8	Generalized	Isolated	Idiopathic	THP 20 mg	No
3	M/18	Segmental	Isolated	Idiopathic	THP 24 mg; BTX	THP 24 mg
4	F/22	Generalized	Isolated	ACTB mutation	THP 16 mg; tramadol 50 mg	THP 12 mg; clonazepam 1.5 mg; clozapine 18.75; BTX
5	F/32	Segmental	Isolated	Idiopathic	Ibuprofen; BTX	No
6	M/9	Generalized	Isolated	DYT-THAP1	THP 21 mg; baclofen 12.5 mg	THP 11 mg
7	M/22	Segmental	Isolated	TTPA	Vitamin E	Unchanged
8	M/47	Generalized	Combined (spasticity)	Cerebral palsy	Antidepressants	Unchanged
9	M/53	Segmental	Isolated	Idiopathic	Clonazepam 0.5 mg; BTX	BTX
10	F/65	Segmental	Combined (parkinsonism)	Idiopathic	Pramipexole; L-dopa; Diazepam 5 mg; BTX	Pramipexole; L-Dopa
11	F/48	Generalized	Isolated	ACTB mutation	THP 12 mg; clozapine 12.5 mg; oxazepam 10 mg; diclofenac; BTX antidepressant	THP 12 mg; clozapine 12.5 mg; antidepressant
12	F/63	Segmental	Isolated	Idiopathic	Clonazepam 2.5 mg	Clonazepam 0.5 mg
13	M/62	Segmental	Isolated	Idiopathic	BTX	Clonazepam 1.0 mg; BTX
14	F/8	Generalized	Combined (spasticity)	Cerebral palsy	THP 1.5 mg; baclofen 12 mg; gabapentin 600 mg; clonazepam 0.5 mg	Unchanged
15	F/63	Segmental	Isolated	Idiopathic	No	No

ACTB: beta-actin gene; BTX: botulinum toxin injections; THP: trihexiphenidyl; TTPA α-tocopherol transfer protein – vitamin E.

### Individual priorities

The 45 priorities (3 per patient) were categorized in self-care/activities of daily living (ADL) (n = 10); comfort in sitting and sleep (n = 9); communication (n = 7); social/leisure activities (n = 7); and mobility (n = 12). Communication priorities involved the ability to use an electric communication device, sign language or normal social interaction without interference of dystonic posturing. Social activities included sports, interactive games or going out for dinner. Mobility comprised walking, cycling, driving a car or the use of public transport.

For each patient, priorities comprised at least two categories. There was a very strong correlation between performance and satisfaction scores (ρ = 0.86, p < 0.0001) and both scores significantly improved after the application of DBS (Table 2). At patient level, a clinically significant change in satisfaction in two or three individual priorities was reported in 73% (11) of the patients. In 47% all three priorities were improved, in 27% two priorities were improved, in 13% one priority was improved and in 13% none of the priorities was improved.

**Table 2:** Pre- and postoperative COPM scores for all functional priorities and per subcategory.

	COPM-Performance			COPM-Satisfaction		

	Baseline	1 year	Improved priorities†	Baseline	1 year	Improved priorities†

All priorities	3.0 (1.0–4.0)	7.0 (5.0–8.0)	32/45*	2.0 (1.0–3.5)	7.0 (4.0–8.5)	31/45*
Sitting and sleep	3.0 (2.0–4.0)	7.0 (5.5–8.0)	8/9	2.0 (1.5–3.5)	7.0 (3.5–9.0)	5/9
Self-care/ADL	1.5 (1.0–4.3)	6.0 (2.5–7.3)	6/10	1.5 (1.0–3.0)	6.5 (2.5–7.3)	7/10
Communication	4.0 (3.0–4.0)	8.0 (6.0–10.0)	5/7	3.0 (1.0–4.0)	9.0 (7.0–9.0)	6/7
Social/leisure	3.0 (1.0–4.0)	7.0 (3.0–7.0)	4/7	3.0 (1.0–4.0)	6.0 (1.0–7.0)	4/7
Transfer	2.5 (1.3–4.8)	6.5 (5.3–7.0)	9/12	2.0 (1.0–3.8)	6.5 (5.3–8.8)	9/12

ADL activities of daily living; † Change or 2 point or more between baseline and 1-year post-operative score. * p < 0.0001.

### Dystonia severity

BFMDRS scores improved with a median change of 30% (pre 46.8 IQR 17.0–66.0 vs post 35.4 IQR 11.3–53.0; p = 0.027). For absolute changes, see supplementary table 1. Eight patients (53%) were classified as responders with a decrease in their BFMDRS of more than 20% and seven (47%) as non-responders.

The non-responders were two patients with cerebral palsy (case 8 and 14), one patient with a mitochondrial disorder (case 1), one patient with DYT-THAP1 (case 6) and three patients with segmental dystonia (case 3, 12 and 15).

### Priorities versus dystonia severity

Change in dystonia severity did neither correlate with change in performance (ρ = –0.15, p = 0.601) nor satisfaction score (ρ = 0.17, p = 0.557).

Seven of the eight responders reported a clinically significant improvement in performance and satisfaction on at least two or three individual functional priorities. In the group of non-responders, despite the lower motor response, clinical significant improvement in at least two priorities was achieved in four of these patients for performance and three for satisfaction, with a statistically significant change in COPM score (Case 6, 12, 14 and 15, p = 0.017).

## Discussion

This prospective case series aimed to systematically evaluate the effectiveness of GPi-DBS as measured with change in preoperatively set functional priorities. The priorities of the patients and their caregivers lay within the domains of ADL, seating and sleep, communication, social/leisure activities and mobility. A clinically significant motor response coincided with improvements in functional priorities in 7/8 patients. Interestingly, half of the motor ‘non-responder’ patients also showed a clinically significant change in two or three priorities. Our findings are in line with a previous study in childhood dystonia showing that DBS may lead to improvement of functional goals also in patients with only moderate to ‘insignificant’ motor response [8].

In contrast to the vast majority of efficacy studies primarily focusing on motor response, we evaluated effect of GPi-DBS by looking at functional priorities. These priorities provide an unique insight in what patients and their caregivers identify as most important aspects in daily living. Given the heterogeneous nature of dystonia, it is not surprising that needs varied greatly between patients. An additional advantage is that this method may facilitate recognition of patients that might be unsuitable for the procedure due to goals that are unrealistic or not likely to be achieved by GPi-DBS. One might argue that with a goal-oriented approach changes are subjective to the patients’ perception of improvement rather than objective symptom reduction. In addition, a potential placebo effect cannot be excluded in the absence of a control group. However, we agree with Kubu and colleagues that the main goal of DBS is to improve quality of life as perceived by the patient more than by the clinician, and that the effect of an elective neurosurgical option as DBS should be measured accordingly [9]. In the future, it would be useful to objectify the patient centered outcome. This can be done by transforming the patients’ priorities into a treatment goal and pre-operatively decide with the patient and caregivers when the goal is met, for instance by using the goal attainment scale.

The heterogeneous patient sample may be seen as a limitation, both in terms of age as well as etiology. On the other hand, it can be seen as an advantage for the generalizability of the study. We did not correct for changes in medication, which could account for some of the perceived improvements. We realize that our conclusions are bases on a small case series with a possibly limited power, but hope these results serve as a pilot study to trigger future studies focusing on the effectiveness of GPi-DBS in dystonia. First to assess to what extent a good motor outcome corresponds with the perceived outcome on the patient’s priorities. This may not always be the case, as 1/8 motor responders did not reach a significant improvement on his priorities, and might provide clarity in the repeatedly reported discrepancy between motor outcome and patient reported outcome. A systematical use of patient centered outcomes might shine a new light on the current opinion that GPi-DBS is more effective in isolated than in combined forms of dystonia.

In conclusion, the effect of GPi-DBS should be measured not by motor symptom reduction alone, as clinically significant improvements on individual predefined priorities can be achieved irrespective of motor response. In addition, a goal- or patient-oriented approach provides unique insights in the priorities in daily living of dystonia patients and their caregivers. This may not only be of added value for DBS candidates, but also for patients across the entire dystonia population.

## Appendix

**Supplementary Table 1:** Absolute Burke-Fahn-Marsden Dystonia Rating Scale score pre- and postoperatively (n = 15).

Pt	Gender/age (yr)	Distribution	Isolated or combined	Mean BFMDRS-M*	

				Pre-DBS	Post-DBS

1	M/8	Generalized	Combined	66	70
2	M/8	Generalized	Isolated	55	31
3	M/18	Segmental	Isolated	47	44
4	F/22	Generalized	Isolated	81	33
5	F/32	Segmental	Isolated	33	13
6	M/9	Generalized	Isolated	71	69
7	M/22	Segmental	Isolated	14	5
8	M/47	Generalized	Combined	49	53
9	M/53	Segmental	Isolated	23	14
10	F/65	Segmental	Combined	16	8
11	F/48	Generalized	Isolated	64	45
12	F/63	Segmental	Isolated	17	18
13	M/62	Segmental	Isolated	20	11
14	F/8	Generalized	Combined	102	107
15	F/63	Segmental	Isolated	9	10

* Mean BFMDRS-M was calculated from the two scores of the experts. BFMDRS-M: Burke-Fahn-Marsden Dystonia Rating Scale, Motor subcale; DBS: Deep brain stimulation.

## Author Information

H Eggink received a MD/PhD bursary from the University Medical Center Groningen and a Ter Meulen grant (KNAW), and travel grants from COST Dystonia Europe, the Dutch Child Neurology Association (NVKN) and the Movement Disorder Society (MDS). JC van Zijl received a MD/PhD bursary from the University Medical Center Groningen and a research support fund from the Dutch Parkinson Society. MF Contarino is on the advisory board of and an independent consultant for research and educational issues for Medtronic. She received speaking fees from Novartis Pharma BV (CME activity). Received a grant from the Stichting Parkinson Fonds. The DBS center of the Haga Teaching Hospital/LUMC received compensation for DBS training activities and an unrestricted educational grant from Medtronic. KJ Peall is an MRC Clinician-Scientist Fellow (MR/P008593/1). MA Tijssen is funded by STW Technology Society–NeuroSIPE, Netherlands Organization for Scientific Research–NWO Medium, Fonds NutsOhra, Prinses Beatrix Fonds, Gossweiler Foundation, Phelps Stichting, Stichting wetenschapsfonds dystonie vereniging, and educational grants from Ipsen, Allergan, Merz, Actelion, and Medtronic.
